# Tuméfaction indolore du pied chez le diabétique: penser au pied de Charcot

**DOI:** 10.11604/pamj.2015.21.15.6732

**Published:** 2015-05-07

**Authors:** Imène Boukhris, Ines Kechaou

**Affiliations:** 1 Service de Médecine Interne B, Hôpital Charles Nicolle, Tunis, Tunisie

**Keywords:** Pied, diabète, ostéoarthropathie nerveuse, foot, diabetes, nerve osteoarthropathy

## Image en medicine

L'ostéoarthropathie de Charcot est une complication grave qui touche les patients diabétiques présentant une neuropathie périphérique. La phase aiguë est rarement identifiée. Seul un diagnostic précoce peut limiter l’évolution vers des déformations du pied, voire l'amputation. Un patient âgé de 58 ans, était hospitalisé pour une suspicion de thrombose veineuse du membre inférieur droit devant l'apparition depuis dix jours, d'une tuméfaction et d'un œdème du pied, non douloureux, avec une difficulté de chaussage. Il avait des antécédents de diabète type 2 depuis 20 ans, insulino nécessitant au stade de complications dégénératives dont une neuropathie périphérique distale des membres inférieurs, confirmée par l’électromyogramme. L'examen trouvait un œdème du dos du pied, un affaissement de la voute plantaire, sans signes inflammatoires locaux. Les pouls pédieux étaient faibles. Ce patient n'avait pas de notion de traumatisme récent. Un érysipèle était peu probable devant l'absence de fièvre, d'hyperleucocytose et de SIB. Une thrombose veineuse était fortement suspectée mais le doppler veineux était normal. Le diagnostic du pied de Charcot était confirmé par la radiographie montrant des luxations et des fractures avec déplacements des têtes des métatarsiens des 2^ème^, 3^ème^ et 4^ème^ rayons, une lyse osseuse de la tête et de la base du 1er métatarsien. Le patient était adressé en médecine physique pour un chaussage orthopédique adapté. L’évolution était marquée par la survenue 3 mois plus tard d'un mal perforant du pied droit nécessitant l'amputation, survenant sur un pied totalement déformé, avec à la radiographie présence de fractures déplacées et des géodes des têtes métatarsiennes.

**Figure 1:**
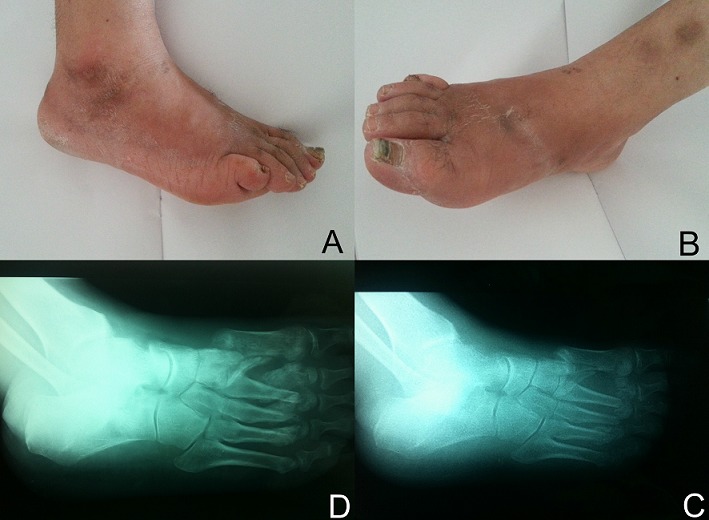
A) photo de profil du pied droit; B): photo de face du dos du pied droit; C): radiographie de profil du pied droit montrant des fractures des têtes des métatarsiens-2^ème^, 3^ème^ et 4^ème^ rayons et une lyse osseuse de la tête et de la base du 1er métatarsien; D) radiographie de profil du pied droit montrant des fractures déplacées, des luxations articulaires et des géodes sous-chondrales des têtes métatarsiennes

